# Estimating Genetic Variance in Life-Span Response to Diet: Insights From Statistical Simulation

**DOI:** 10.1093/gerona/glac172

**Published:** 2022-08-26

**Authors:** Alistair M Senior

**Affiliations:** Charles Perkins Centre, University of Sydney, Sydney, New South Wales, Australia; School of Life and Environmental Sciences, University of Sydney, Sydney, New South Wales, Australia; School of Mathematics and Statistics, University of Sydney, Sydney, New South Wales, Australia

**Keywords:** Animal model, Dietary restriction, Mortality, Power analysis, Quantitative genetics

## Abstract

Several studies demonstrate genetic variation in response to dietary restriction (DR) by replicating treatments across isogenic lines/strains from genetic reference panels. These studies typically quantify the response to DR as an effect size, estimated for each strain separately (eg, the difference in mean life span between groups). Such “no-pooling” analyses are expected to systematically overestimate variation in response DR, principally by overlooking sampling variance. In contrast, “partial-pooling” analyses using mixed-effects models are less prone to this bias. I demonstrate these issues using simulations, which also show that partial-pooling analyses can improve replicability among studies. Regardless of the analyses used, estimates of among-strain variation will have low precision when sample sizes are small. A worked example using survival data in mice is given. Life-span studies using genetic reference panels always have to trade-off within- and among-strain replication owing to logistical challenges. The simulation presented can also be used to help design such studies through power analysis.

Dietary restriction (DR; sometimes also termed caloric restriction) involves a reduction in the calories consumed while avoiding malnutrition. Relative to ad libitum (AL) feeding, DR extends life in numerous organisms (1,2). However, DR does not universally improve life span for all individuals (eg, (3,4)) and genetic background likely contributes to some of this variation.

Genetic reference panels constitute dozens of lines of a model organism, where the genetic variation within each line has been minimized, usually through inbreeding (eg, (5,6)). Collectively, the lines are designed to provide a picture of the total functional genetic variation in the outbred parent population. By replicating experiments en masse across lines, one can estimate genetic variation in response to a treatment like DR. Such studies do not normally involve a prediction for the sign or magnitude of effect in any one specific strain. Rather, the different strains are expected to vary randomly and to differing degrees from one another, and the aim is to quantify such variation.

Several studies have reported among-strain variance in response to DR using genetic reference panels (see ref. (7)). Such conclusions are often supported by “waterfall plots,” which display pairwise effect sizes (often difference in mean life span) for each strain. Presenting variability among strains in this way, however, conflates sampling and biological variation, resulting in overestimation of variation among strains. Unfortunately, sampling variance is often high in genetic reference panel studies on life span where sample sizes per strain can be small. For example, Liao et al. (8) compared 40% DR to AL in ~40 ILSXISS strains of mice, with around 5 animals per strain per sex per treatment. Based on the variation in the pair-wise effect sizes for each strain, they concluded that there is “marked genetic variation among strains” in response to DR. Subsequently, Rikke et al. (9) applied 40% DR to many of the same strains using 10–12 females per strain per diet. They again found among-strain variation in the response. However, the responses of several strains were not replicated and in some cases were even reversed (eg, strains 115, 117, and 122).

Here, I use statistical simulation to show how calculating individual pairwise effect sizes, also known as a “no-pooling” analysis (10), risks overestimating genetic variance in responses to DR, and compromises between-study replicability of genetic effects. I show that this problem is exacerbated when sample sizes are small and life-span data are treated as though normally distributed. I show how alternative “partial-pooling” analyses using mixed-effects models (11,12) minimize these issues. Mixed-effects models are explicitly designed to quantify variation in responses among different clusters/groups (eg, different isogenic strains) within a population. Variants of the analyses based on principles of time-to-event analysis can further minimize biases. Finally, I present a worked example of such an analysis in rodents. All code has been made available to facilitate uptake and can be readily used to design future studies.

## Method

### Simulation

All analyses were implemented in R. Code is available at https://github.com/AlistairMcNairSenior/GenRefPanelSimulation.

Survival data were simulated using the “simsurv” function in the *simsurv* package (13). The *simsurv* package allows the user to simulate data from several statistical distributions commonly used to model survival data. For the current simulation, *simsurv* is particularly useful as it allows the user to simulate clustered effects, such as the strain-specific effects that might be seen in a genetic reference panel study.

**Figure 1.**
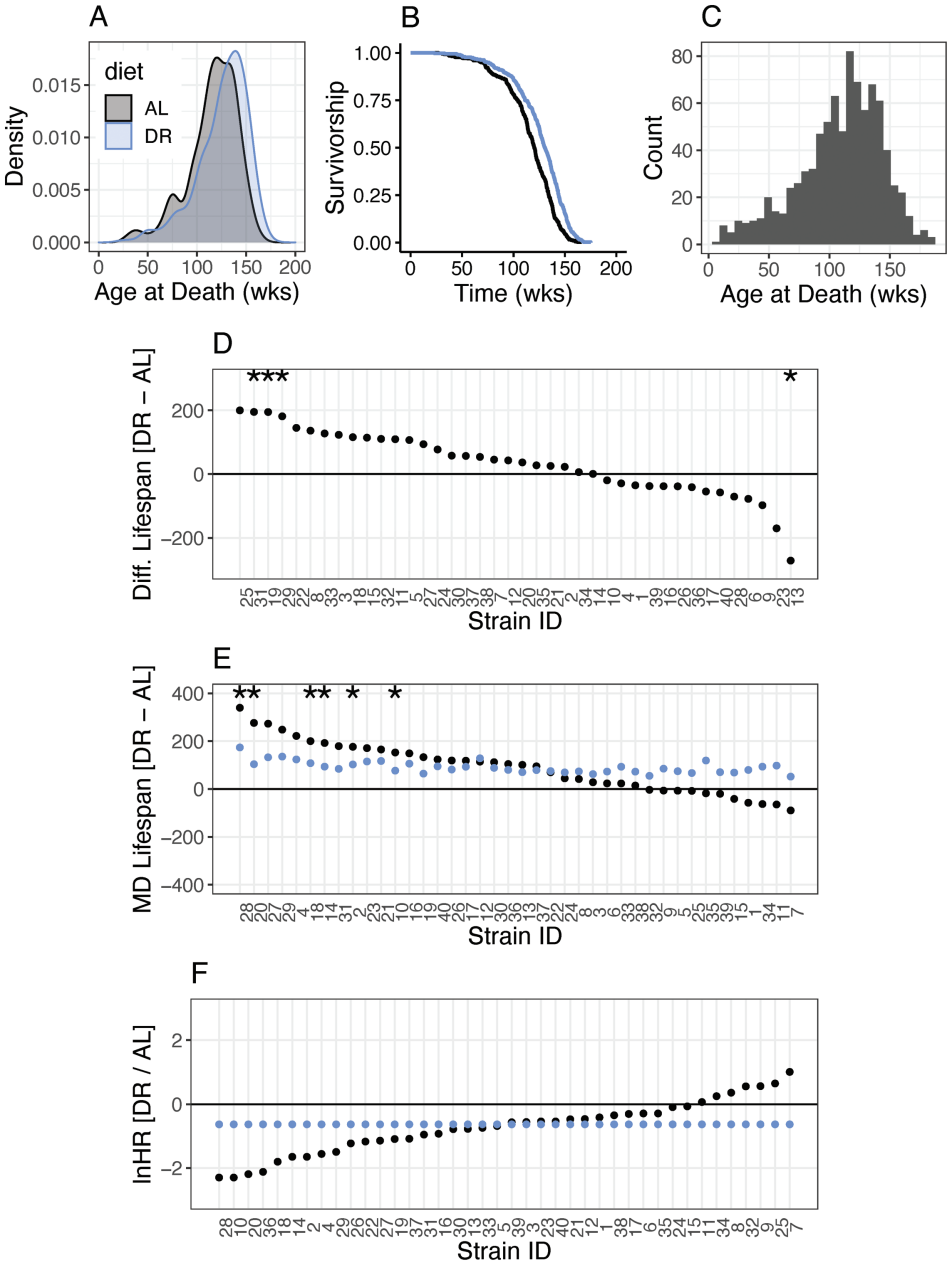
(**A**) Distribution of the age-at-death for 400 simulated animals of the same strain under AL (black) and DR (blue). (**B**) Kaplan–Meir plot for the survival of data in (A). (**C**) Frequency histogram of the ages of death of female ILSXISS mice; data taken from Rikke et al. (9). (**D**) Waterfall plot showing the simulated effect of DR in 40 different strains (5 animals per strain per diet) as quantified by mean difference in the age at death (MD) estimated for each strain separately (ie, no pooling). * indicates a statistically significant difference in mean survival between DR and AL within the strain. (**E**) Replicate simulation of C, including MD estimated via LMM (ie, partial pooling; blue). (**F**) Effects of DR as quantified by the lnHR estimated using CM (ie, no pooling; black points) and CMM (ie, partial pooling; blue points). For all simulations, the true overall lnHR for DR/AL is −0.5 and there is no genetic variance in response to DR. AL = ad libitum; CM = Cox proportional-hazards model; CMM = Cox proportional-hazards mixed-effects model; DR = dietary restriction; LMM = linear mixed model; MD = mean difference.

In many organisms, the risk of mortality increases with age, meaning that the distribution of ages at death is skewed (ie, non-normal). In these simulations, I use a Gompertz distribution, a popular distribution that assumes that the hazard of mortality increases exponentially with age as:


$$\begin{array}{l} h\left(t\right)=a\times e^{bt} \end{array}$$ (1)

where *h*(*t*) is the hazard of death at time *t*, *a* is the baseline hazard, and *b* is the time-dependent change in the hazard. The Gompertz distribution has been widely used to model mortality in rodents, meaning the literature can be used to parameterize this simulation. Based on Simons et al. (14), estimates of the typical *a* and *b* in AL-fed rodents are log(*a*) = −11.57 and log(*b*) = −4.9. Assuming these parameters gives a slightly skewed distribution, with a heavy negative tail and the bulk of mortality beyond 100 weeks (black group; Figure 1A and B). This pattern of survival is comparable to that seen in real mice (Figure 1C).

I simulated an experimental design where replicate animals from several genetic strains were subjected to both AL and DR. The hazard for animal *i* in strain *j* at time *t* was given by:


$$\begin{array}{l} h{\left(t\right)}_{ij}=h\left(t\right)\times e^{X_{i}(\mu +\delta_{j})} \end{array}$$ (2)

$$\begin{array}{l} \delta_{j}∼N\left(0,\;\sigma_{\delta }\right) \end{array}$$ (3)

where *h*(*t*) is the base hazard coming from eqn (1), *X*_*i*_ is a dummy predictor stating whether animal *i* is under DR or not (0 = AL, 1 = DR), *μ* is the overall natural logarithm of the hazard ratio (lnHR) for the effect of DR, and *δ*_*j*_ is the deviation of strain *j* from the overall *μ*. Strain-specific effects were drawn from a normal distribution with a mean of 0 and standard deviation (*SD*) *σ*_*δ*_ (eqn (3)).

In this model, the parameter for the among-strain (genetic) variation in the effect of DR on survival is *σ*_*δ*_ (*SD* of strains around mean effect, *μ*). A useful metric for the degree of variation in response is the coefficient of genetic variation in response, CV_*G*_:


$$\begin{array}{l} CV_{G}=\frac{\sigma_{\delta }}{\left|\mu \right|} \end{array}$$ (4)

CV_*G*_ is useful because it is unitless and thus facilitates comparisons among estimators.

Figure 1A and B shows simulated survival data for 400 animals of the same strain under DR (blue) and AL (black), assuming lnHR, *μ*, is −0.5 (the average effect of DR across taxa, but a conservative value in rodents (1)). The median life span is extended under DR by 8.6% from 842 days to 914 days (AL vs DR mean and modal life span = 813 vs 881 and 937 vs 1 004).

This simulation assumes homogenous survival among strains under AL. In the Supplementary Text 1, I present an extended model that includes genetic variance in survival in the AL group. However, this model is more complex and simulations from it do not yield different conclusions from those in the main text.

### Statistical Models

I began by quantifying the effect of DR using the difference in mean life span between DR and AL (mean difference [MD]) as an effect size; MD has been widely used (eg, (8)). To calculate CV_*G*_, I estimated the *SD* among strains (*σ*_*δ*_ in eqn (4)) in MD using 2 approaches.

The first was a “no-pooling” (or “fixed-effect”) analysis, where the effect sizes were estimated by applying a linear model (LM) to data stratified by strain, before calculating the *SD* among these estimates. As a visualization of the estimated variation among MDs, I used the waterfall plots that have been routinely presented and interpreted as evidence of genetic variability elsewhere (eg, reconstituted in (7)). The no-pooling approach provides a separate (ie, unpooled) statistical test for each strain, allowing the user to state whether the MD for any given strain is “statistically significant.” However, this approach is expected to overstate the variability among MDs because it conflates biological variance (ie, differences between strains) and sampling variance (ie, variation arising due to sampling alone).

Second, I applied a “partial-pooling” (or “random-effect”) analysis using a single linear mixed model (LMM). Internally, the LMM uses random regression, where the MD for each specific strain is approximated as the weighted average of the MD estimated from strain-specific data and the MD from the data for all strains pooled (10). Because the sample size (and thus weight) for each individual strain is lower than that for all data combined, the strain-specific MDs are pulled toward the pooled MD on which they are centered. This effect, termed “shrinkage,” limits the variability among strain-specific MDs. These partial pooling analyses are not designed to test for the presence of a statistically significant effect in one strain or another. However, because of shrinkage, partial pooling is expected to result in a less upward bias in the estimated variation among strains than the no-pooling equivalent.

LMs and LMMs assume that (the residuals of) life-span data are normally distributed. While larger sample sizes can provide some protection, violating the assumption of normality can be particularly problematic when sample sizes are small as is often the case for life-span studies on rodents. An alternative approach based on time-to-event principles is to estimate the lnHR. To estimate variation in lnHR, I used a Cox proportional-hazards model (CM) applied to each strain and a Cox proportional-hazards mixed-effects model (CMM; a type of “frailty model”). Here, the CM represents a no-pooling analysis, while the CMM is the partial pooling equivalent.

LMs were implemented using the “lm” function in *base* and CMs using the “coxph” function in the *survival* package. LMMs and CMMs were implemented using the “lmer” and “coxme” functions in the *lme4* (15) and *coxme* (16) packages, respectively.

## Results

### Simulation

Figure 1D shows the results of a simulated 40-strain study, with 5 animals per diet per strain. The parametrized effect of DR was −0.5, which was homogeneous among strains (CV_*G*_ = 0). The no-pooling MD effect sizes for the different strains range from life-span extension of 200 days to a reduction of 271 days. There are statistically significant increases in 3 strains, and a significant reduction in one. A crude visual interpretation of the range of effect sizes in Figure 1D, coupled with the statistical significance of some and not others, would lead one would conclude that there is substantial genetic variation among strains where DR extends life in some but decreases life in others.

Demonstrating the sensitivity of the analysis to sampling, in Figure 1E I have re-simulated the same experiment. This time there are significant increases in mean life span in several strains and no significant decreases. The mean effect size shown in Figure 1E is +90.8 days; the *SD* among effects is 106.1 days. Hence the estimated CV_*G*_ is 1.17, despite the true value being 0. This analysis again suggests genetic variation in response. However, it is notable that the rank order of strains has changed from that in Figure 1D highlighting that the genetic effects found previously are not repeatable.

Re-analyzing the same data in Figure 1E using an LMM with partial pooling suggests less variation. The overall effect estimated by the LMM is again +90.8 days, but the estimated *SD* among strains is just 42.7, making the CV_*G*_ 0.47 (ie, closer to the true value of 0). For a visual comparison of the estimated strain-specific effects from the pooling analysis (ie, with shrinkage), I have overlaid the “strain-specific” estimates from the LMM in Figure 1E (blue series).

Figure 1F is based on the same data as Figure 1E but with effects quantified via the lnHR. The no-pooling analysis (black series) using the CM again suggests substantial inter-strain variation. However, the CMM (blue series) suggests no variation. From the CMM, the overall estimate of the lnHR is −0.63 (true value = −0.5), the estimated *SD* among strains is 0.008 and the CV_*G*_ is 0.014 (true value = 0).

**Figure 2.**
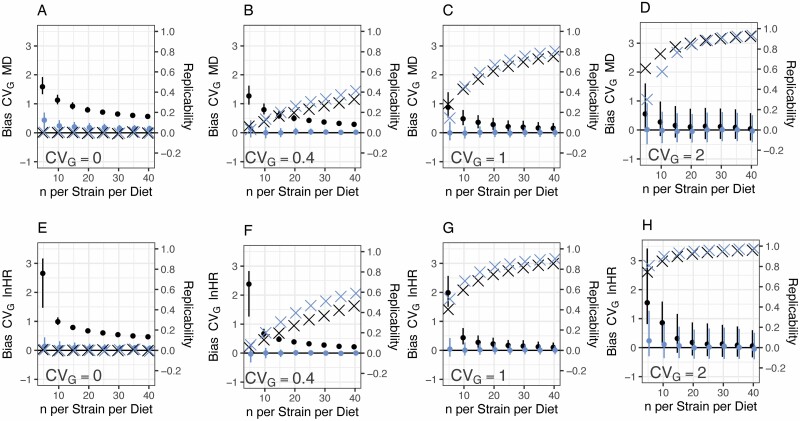
Bias in the estimated CV_*G*_ (solid points) and inter-experiment replicability of strain-specific effects (crosses) as a function of sample size per strain per group and among-strain variation. Black points use estimation with no-pooling (LM or CM), while blue points use partial pooling (LMM or CMM). (**A–D**) uses the MD effect size, while (**E–H**) use the lnHR. For bias, the median ± inter-quartile range of 1000 replicate simulations is presented. Replicability is the mean correlation between 1000 pairs of experiments in the same genetic strains. For all simulations, the true overall lnHR for DR/AL is −0.5, the assumed genetic variance in response to DR is as shown on the panel, and 40 strains per experiment were simulated. AL = ad libitum; CM = Cox proportional-hazards model; CMM = Cox proportional-hazards mixed-effects model; DR = dietary restriction; LM = linear model; LMM = linear mixed model; MD = mean difference.

No-pooling methods become less biased as the sample size increases regardless of the degree of true variation (Figure 2). However, this approach will still estimate the substantial genetic variance in response to DR when none exists, even with sample sizes as high as 40 animals per strain per diet (Figure 2A and E; black series). In contrast, partial-pooling analyses display less bias (Figure 2A and E, blue series). As the amount of among-strain variance present increases, biases in the CV_*G*_ from no-pooling methods become less extreme but are still present (Figure 2). It is notable that where there is variation present, small sample sizes can generate a lot of variation in the estimated CV_*G*_ regardless of the method used (width of error bars; Figure 2, *n* = 5).

I estimated the replicability of strain-specific effects of DR (ie, strain-wise correlation of effects in 2 experiments: crosses, Figure 2). In most cases, partial pooling analyses improved replicability (blue vs black crosses, Figure 2). The CMM outperformed other methods for replicability (Figure 2).

Finally, I used the simulation to estimate the sample size needed to detect statistically significant among-strain variation in response to DR using the CMM. In a 40-strain study, where the CV_*G*_ was 0.4, the power to detect significant among-strain variance was 80% at 35 animals per strain per diet. Where the CV_*G*_ was 1, power rose above 80% at between 5 and 10 animals.

### Re-analysis

**Figure 3.**
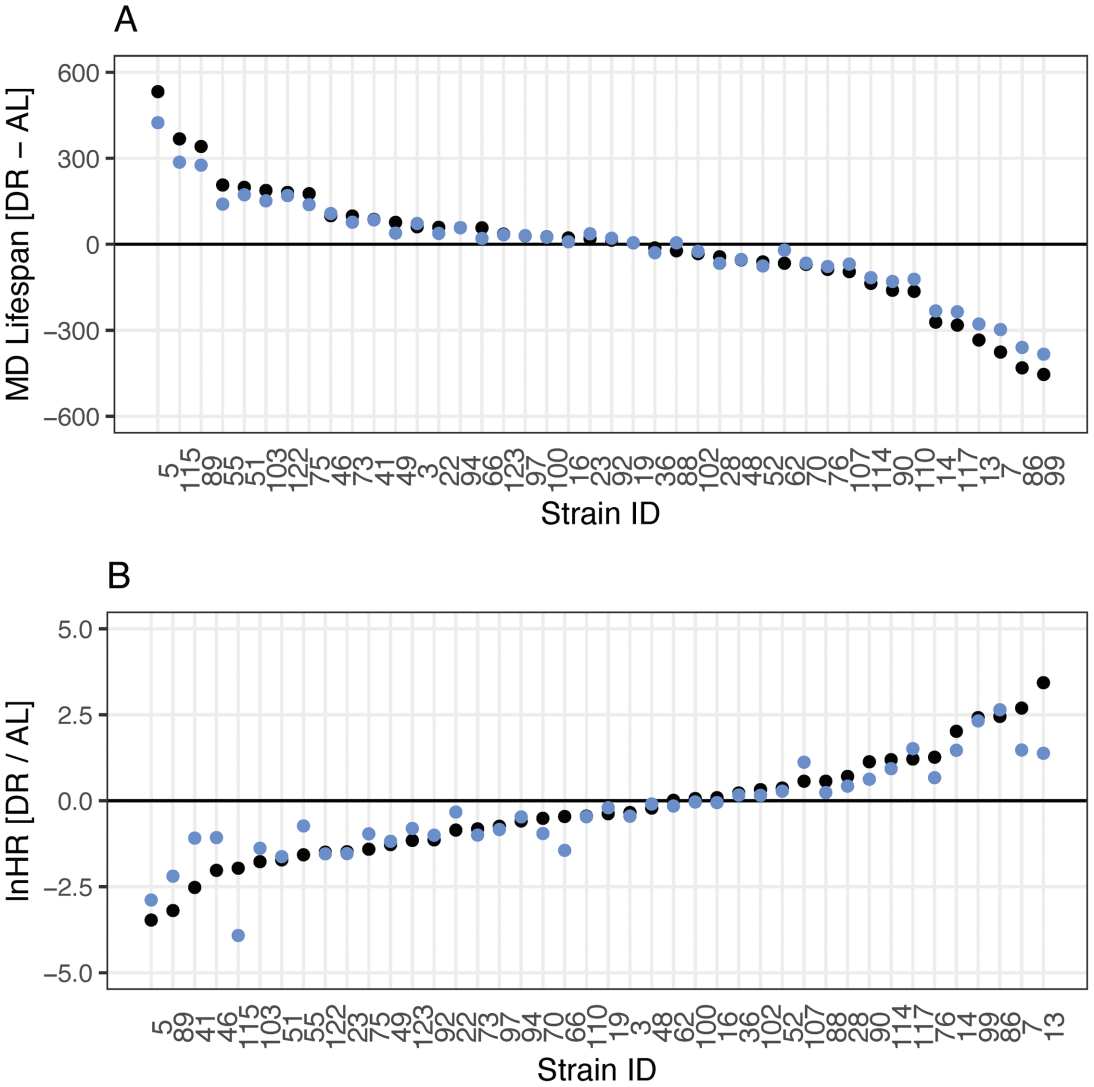
(**A**) Effects of DR on life span in female ILSXISS mouse strains as quantified by the MD using no-pooling (LM; black) and partial pooling (LMM; blue). (**B**) Effects of DR on life span in female ILSXISS mouse strains as quantified by the lnHR using no-pooling (CM; black) and partial pooling (CMM; blue). Data taken from Rikke et al. (9). AL = ad libitum; CM = Cox proportional-hazards model; CMM = Cox proportional-hazards mixed-effects model; DR = dietary restriction; LM = linear model; LMM = linear mixed model; MD = mean difference.

I re-analyzed the survival data from Rikke et al. (9) quantifying the effect of DR *via* MD and lnHR estimated with no and partial pooling (Figure 3; see Supplementary Text 2 for full details and results). For most strains, the partial-pooling estimates are more conservative than the no-pooling equivalent. For the MD effect sizes, the CV_*G*_ is 39.3 with no pooling and 35.4 with partial pooling. For the lnHR, the CV_*G*_ is 5.96 with no pooling and 4.39 with partial pooling. In this case, while the partial-pooling estimates suggest less variability among strains than their no-pooling equivalents, there is still substantial variation estimated. Further, tests for improvement in fit of the LMM and CMM both suggest the among-strain variance in response to DR is statistically significant (Supplementary Text 2).

## Discussion

Genetic reference panel experiments can be used to estimate genetic variation in response to DR. However, these studies need to be appropriately powered and use suitable analytical methods. Analyses using a no-pooling approach risk substantially overestimating genetic variation in response to DR, especially where sample sizes are small. Mixed-effects models, which use partial pooling, are less prone to this bias.

Re-analyzing the data from Rikke et al. (9), I found that partial-pooling analyses reduced the amount of among-strain variation in response to DR. Even so, the estimated genetic variability was still high. It seems unlikely that the application of no-pooling analyses alone explains the high variation and low replicability of response to DR in ILSXISS mice across studies (8,9). One possibility is that the low sample sizes of these studies have generated imprecise estimates. As I show, with 5–10 animals per strain per diet, even partial-pooling analyses have low precision when there is variation present. Supporting this argument is another recent and more powerful experiment (30 animals per strain per diet) in just 8 strains (17). This more “precise” study also failed to replicate key observations in Liao et al. (8). Of course, undescribed differences in housing, micro-environmental changes, or even maternal effects (usually handled via the analysis of half-siblings) could also have caused contrasting results between studies. These study-wise specificities likely underlie a broader issue of replication in preclinical research (18).

Balancing among- versus within-strain replication in genetic reference panel studies is a logistical challenge. Increasing the number of strains while keeping sample sizes low may improve the ability to estimate the breadth of among-strain variability but means that the replicability of specific strains will be compromised (and vice versa). The simulation presented can be used as a powerful analysis tool to help navigate these issues in study design (Supplementary Text 3).

## Supplementary Material

glac172_suppl_Supplementary_Material
