# Supplementary material for: Estimating Genetic Variance in Life-Span Response to Diet: Insights From Statistical Simulation
Source: J Gerontol A Biol Sci Med Sci. 2022 Aug 26;78(3):392–6. doi: 10.1093/gerona/glac172 (PMC9977232; doi:10.1093/gerona/glac172)
Supplement: glac172_suppl_Supplementary_Material [file glac172_suppl_supplementary_material.pdf]

Appendix for Estimating genetic variance in lifespan response to diet: insights from statistical simulation

Alistair M. Senior PhD[1,2,3,*]

1. Charles Perkins Centre, University of Sydney, NSW 2006, Australia

2. School of Life and Environmental Sciences, University of Sydney, NSW 2006 Australia

3. School of Mathematics and Statistics, University of Sydney, NSW 2006 Australia

* Correspondence: alistair.senior@sydney.edu.au

eText1: Extended Model

The simulation in the main text assumes that baseline hazard of different strains on the *ad libitum* (AL) regime is homogenous. An alternative model that does not make this assumption gives the hazard for an animal *i* in strain *j* at time *t* as:

$$h(t)_{ij} = h(t) \times e^{\gamma_j + X_i(\mu + \delta_j)} \quad (1),$$

$$\begin{bmatrix} \gamma_j \\ \delta_j \end{bmatrix} \sim N\left(\begin{bmatrix} 0 \\ 0 \end{bmatrix}, \begin{bmatrix} \sigma_\gamma^2 & \sigma_{\gamma\delta} \\ \sigma_{\gamma\delta} & \sigma_\delta^2 \end{bmatrix}\right) \quad (2),$$

$$\sigma_{\gamma\delta} = \rho_{\gamma\delta}\sigma_\gamma\sigma_\delta \quad (3),$$

where $h(t)$ is the baseline hazard coming from eqn. 1 in the main text, γ_j is the deviation of strain *j* from the baseline hazard under the AL condition, X_i is a dummy variable stating whether animal *i* is under DR or not (0 = AL, 1 = DR), μ is the overall mean log hazard ratio (lnHR) for the effect of DR, and δ_j is the deviation of strain *j* from the overall μ. The strain-specific effects, γ_j and δ_j are drawn from a bi-variate random-normal distribution with means of 0, standard deviations (SDs) σ_γ, and σ_δ (eqn. 2) and covariance $\sigma_{\gamma\delta}$. $\sigma_{\gamma\delta}$ can be more intuitively parametrised from the equivalent correlation, $\rho_{\gamma\delta}$ (eqn. 3).

This model is likely to be more realistic than that in the main text because animals of different strains almost certainly have variable survival under AL conditions (e.g., (1, 2)). However, this model has an additional two parameters; σ_γ (variance among strains on AL) and $\rho_{\gamma\delta}$ (correlation between variance under AL and the response to DR). Regarding, $\rho_{\gamma\delta}$, data from ILSXISS mice suggest that DR extends lifespan in short lived strains but shortens life in long-lived variants (eText 2 and 1, 2). Here I report the results of the main simulation using the extended model assuming such a correlation exists.

eFig. 1 is the equivalent to fig. 2 in the main text, but with simulation the extended model. The patterns within two sets of results are the same: (1) no-pooling analyses overestimate CV_G where partial-pooling is less sensitive, (2) as sample sizes increase biases in no-pooling analyses become less severe, (3) for larger parameterised variances no-pooling analyses become less biased, but overall estimates can be expected to be less precise and (4) replicability is highest for the CMM analysis.

eText 2: Re-Analysis of ILSXISS Data as a Worked Example

I obtained the data on survival of female ILSXISS mice from Rikke, Liao, McQueen, Nelson and Johnson (1) and re-analysed it using the four different methods described in the main text; linear model (LM; no-pooling mean-difference, or MD, based analysis), linear mixed model (LMM; partial-pooling MD based analysis), cox-proportional hazards model (CM; no-pooling lnHR based analysis), and cox-proportional hazards mixed model (CMM; partial-pooling lnHR based analysis). Models were implemented using the same functions as used in the main-text.

The LM and CM analyses apply a separate model to each strain, where the outcomes are respectively, age at death and survival time, with the treatment group as the predictor. The LMM and CMM analyse those same outcomes with all strains collectively. Here the dietary treatment is included as a fixed-effect, and as a random-regression for the strain. These models simultaneously estimate the variance among strains in survival on the AL diet, the response to DR, and yield strain-specific effects using partial pooling. To assess whether the estimated strain-specific variance in response to the treatment was statistically significant (i.e., non-zero), null LMM and CMMs were also implemented. These null models only included a random intercept for the strain, but no random regression; i.e., they assume a homogeneous response to DR. The fit of the null and full random-regression models was compared based on the change in deviance. A statistically significant improvement in model fit is indicative of among-strain variance in response to DR.

The strain-specific estimates from the four analyses are shown in fig. 3 in the main-text. Here I present the complete model estimates, which are described and interpreted in full detail for the interested reader.

The LMM estimates that the average lifespan on an AL diet for a 'typical strain' is 766 days, while the effect of DR in a 'typical strain' is to reduce in mean lifespan of 5 days, which does not differ significantly from no-effect (eTable 1). The SD of individual strains from the ILSXISS panel around these typical estimates is estimated to be 109 and 183 days respectively. The CV_G for the effect of DR is based on this estimated SD and the estimated effect in a typical strain; $CV_G = 183 / |-5| = 36.6$. As a bonus, these models also estimate the correlation between strain survival on the AL treatment and the effect of DR. A negative correlation of -0.42 is estimated suggesting that we see negative effects of DR (i.e., reductions in lifespan) in longer-lived strains and *vice versa*.

The CMM estimates that the lnHR for a typical strain is -0.36 indicating a reduction in mortality on the DR, although the effect is not statistically significant (eTable 1). It is estimated that the SD of strains around the baseline hazard on the AL diet is 0.85, while the SD around the lnHR is 1.36. From the model estimates the $CV_G = 1.36 / |-0.36| = 4.53$. The CMM also estimates a negative correlation between a strain's baseline hazard and lnHR (-0.34), suggesting strains with high mortality on the AL diet, tend to experience larger reductions in mortality under DR.

eTable 2 shows the estimates from the null LMM and CMM, which exclude the strain-specific variance in response to DR. eTable 3 shows the results of a test for improvement in in model between pairs of models. In both cases the improvement in model fit by the inclusion of the among-strain variance in response to DR is statistically significant.

Finally, it is worth noting that a Kaplan-Meir survival curve from all collected data shows that the survival curves for the two diets cross at ~820 days (efig. 2A). This pattern is sometimes interpreted as time dependent effect (i.e., proportionality of hazards is not the same across time). However, a similar pattern can emerge from the heterogenous baseline hazards and hazard ratios estimated by the CMM grouped together (efig. 2B).

eText 3: Power-Analysis by Simulation

The simulation described can be used for power-analysis in study design. To facilitate this, I have written a wrapper function, 'power_sim' for the simulation, which estimates the accuracy (bias in effect sizes), precision (among-simulation variation in effect sizes), power to detect effects and replicability all using the LMM and CMM partial-pooling analyses. Here is a worked example, with code available at https://github.com/AlistairMcNairSenior/GenRefPanelSimulation (script '9.Power_Sim.R').

Hypothetical Example

Let's assume we want to quantify for genetic variance and the effects of DR in a panel of a model rodents relative to AL feeding. The panel comprises 50 lines. Do we need to include all 50 strains from the panel, and how many animals per strain per diet should we use?

Parametrising a Simulation

Based on Simons, Koch and Verhulst (3) we assume that the Gompertz parameters for survival in the AL group are log(a) = -11.57 and log(b) = -4.9. These parameters could also be estimated from survival data directly derived from the panel if such data existed. Also, based on the meta-analysis in Nakagawa, Lagisz, Hector and Spencer (4) we assume that the overall average effect of DR in a 'typical strain' will be a reduction in mortality, such that the lnHR (μ, following notation in eqns 1:3) is -0.5.

The function uses the extended model in eqns 1 through 3, but easily defaults to the simpler model by parameterising σ_γ (among-strain variance in the baseline hazard) and $\rho_{\gamma\delta}$ (correlation among strain-specific baseline hazard and effect of DR) to 0. These parameters could be included if of interest and/or suitable data for estimation are available. The main parameter to be included is then σ_δ, which is the among-strain SD in the effect of DR.

It is recommended that power analysis parametrise the key effect of interest based on the smallest effect that is considered to be of interest (5). For the current case, to select a value for σ_δ, let's assume that smallest level of genetic variance that is of interest is $CV_G = 1$; this is because, based on a *z*-distribution $CV_G = 1$ indicates that the line of no-effect lies 1SD away from the overall mean effect, and that consequently in ~16% of strains there is no-effect of DR or that the effect is in the opposing direction to the average. Given that:

$$\mathrm{CV}_G = \frac{\sigma_\delta}{|\mu|},$$

The minimal value of σ_δ of interest will be:

$$\sigma_\delta = \mathrm{CV}_G \times |\mu| = 1 \times |-0.5| = 0.5.$$

Summarily, based on the arguments above for parametrisation; log(a) = -11.57, log(b) = -4.9, $\mu = -0.5$, $\sigma_\delta = 0.5$, $\sigma_\gamma = 0$, $\rho_{\gamma\delta} = 0$. We are primarily interested accurate estimation of CV_G (parametrised to be 1), as well as ability to detect statistically significant σ_δ.

Results

eFigs 3A and 3B shows the power to detect statistically significant variation in response to DR using MD (based on LMM) and lnHR (based on CMM) respectively. With 50 strains power is above 80% for all sample sizes tested. However, with 30 strains, more than 10

animals are needed for the MD method (eFig. 3A). eFigs 3C and D show the distribution of estimated CV_G for the MD (based on LMM) and lnHR (based on CMM). Increases in the sample size and/or number of strains do not change the accuracy of estimated variation (i.e., there is no systematic bias), however with more animals and strains estimates of CV_G become more precise (i.e., less variable). Finally, eFigs 3E and 3F show replicability of strain-specific effects. For both methods there is a big improvement going from 10 to 15 animals per strain, but diminishing returns beyond 15.

Based on these results, I would argue that assessing 30 strains with 15 animals per strain per diet is an efficient design (total animals = 900). Adding the additional 20 strains does not substantially improve any of the key parameters tested. As a point of contrast, a design with all 50 strains, and 10 animals per strain per diet requires more animals (total animals = 1000), has reduced power and replicability.

An important note is that this simulation assumes that the strains being tested are essentially a random selection of those available and have not been preselected on the basis of a known response or *apriori* hypothesis based on genomic data.

References

1. Rikke BA, Liao CY, McQueen MB, Nelson JF, Johnson TE. Genetic dissection of dietary restriction in mice supports the metabolic efficiency model of life extension. *Exp Gerontol*. 2010;45:691-701.
2. Liao C-Y, Rikke BA, Johnson TE, Diaz V, Nelson JF. Genetic variation in the murine lifespan response to dietary restriction: from life extension to life shortening. *Aging Cell*. 2010;9:92-95.
3. Simons MJP, Koch W, Verhulst S. Dietary restriction of rodents decreases aging rate without affecting initial mortality rate – a meta-analysis. *Aging Cell*. 2013;12:410-414.
4. Nakagawa S, Lagisz M, Hector KL, Spencer HG. Comparative and meta-analytic insights into life extension via dietary restriction. *Aging Cell*. 2012;11:401-409.
5. Albers C, Lakens D. When power analyses based on pilot data are biased: Inaccurate effect size estimators and follow-up bias. *J Exp Soc Psychol*. 2018;74:187-195.

eTable 1

Estimates from the linear-mixed model (LMM) and cox proportional hazard mixed model (CMM) applied to data from female ILSXISS mice. Standard errors (SE), test statistics and p-values are only provided for fixed effects. Test statistics for the LMM are t-values (d.f.) and *z*-values for the CMM. *r* = correlation, MD = mean difference, lnHR = log hazard ratio.

Model	Parameter	Estimate	SE	Statistic	p
LMM	Intercept_{AL}	766.1	19.55	39.19 (41.83)	<0.0001
	MD_{DR-AL}	-5.173	31.22	-0.166 (40.73)	0.869
	$SD_{Int.}$	109.3			
	SD_{MD}	182.7			
	$SD_{Residual}$	196.3			
	$r_{Int., MD}$	-0.420			
CMM	$lnHR_{DR / AL}$	-0.309	0.222	-1.390	0.160
	$SD_{Baseline.}$	0.851			
	SD_{lnHR}	1.360			
	$r_{Baseline, lnHR}$	-0.338			

eTable 2

Estimates from the null linear-mixed model (LMM) and null cox proportional hazard mixed model (CMM) applied to data from female ILSXISS mice. Standard errors (SE), test statistics and p-values are only provided for fixed effects. Test statistics for the LMM are t-values (d.f.) and *z*-values for the CMM. *r* = correlation, MD = mean difference, lnHR = log hazard ratio.

Model	Parameter	Estimate	SE	Statistic	p
LMM	Intercept_{AL}	767.1	20.41	37.59 (57.02)	<0.0001
	MD_{DR-AL}	-8.309	14.74	-0.564 (832.1)	0.573
	$SD_{Int.}$	112.0			
	$SD_{Residual}$	216.4			
CMM	$lnHR_{DR / AL}$	-0.191	0.077	-2.49	0.013
	$SD_{Baseline.}$	0.736			

eTable 3

Significance of change in model fit between full models with random-regression and null models. χ^2 is based on difference in deviance.

Model	Version	Log Likelihood	χ^2	df	p
LMM	Null	-5971			
	Full	-5922	98.91	2	<0.0001
CMM	Null	-4933			
	Full	-4845	174.5	2	<0.0001

eFigure 1

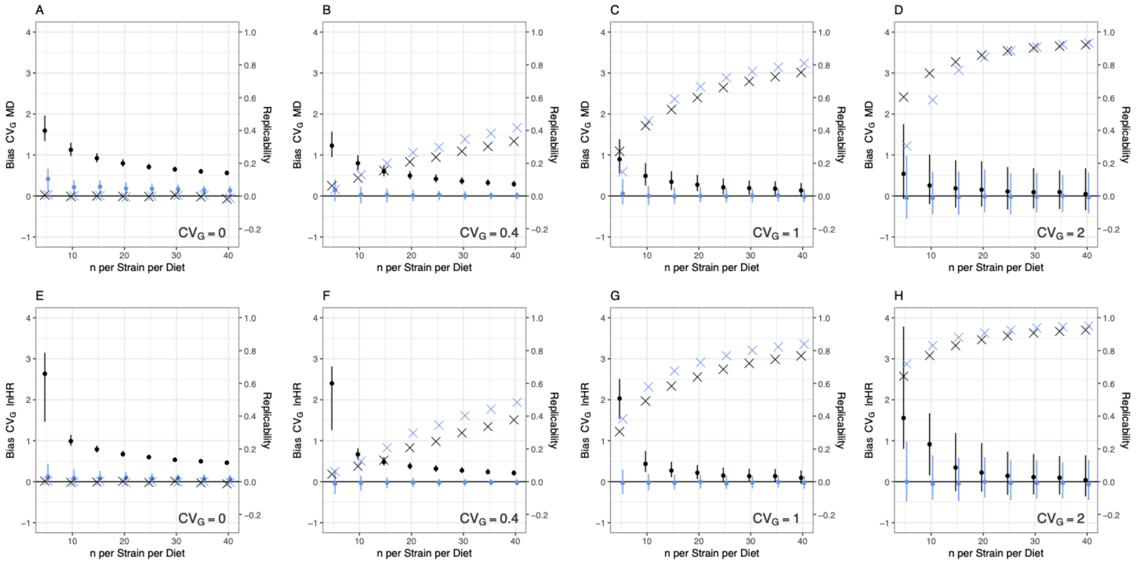

Bias in the estimated CV_G (solid points) and inter-experiment replicability of strain-specific effects (crosses) as a function of sample size per strain per group and among-strain variation. Black points use estimation with no-pooling (LM or CM), while blue points use partial pooling (LMM or CMM). (A-D) uses the MD effect size, while (E-H) use the lnHR. For bias the median ± inter-quartile range of 1000 replicate simulations is presented. Replicability is the mean correlation between 1000 pairs of experiments in the same genetic strains. For all simulations the true overall lnHR for DR/AL is -0.5, the assumed genetic variance in response to DR is CV_G * 0.5, the genetic variance in survival on the AL diet is CV_G * 0.5 / 1.5, the correlation between survival on the AL diet and response to DR is -0.3 (based on the analyses in eText 2), and 40 strains per experiment were simulated.

eFigure 2

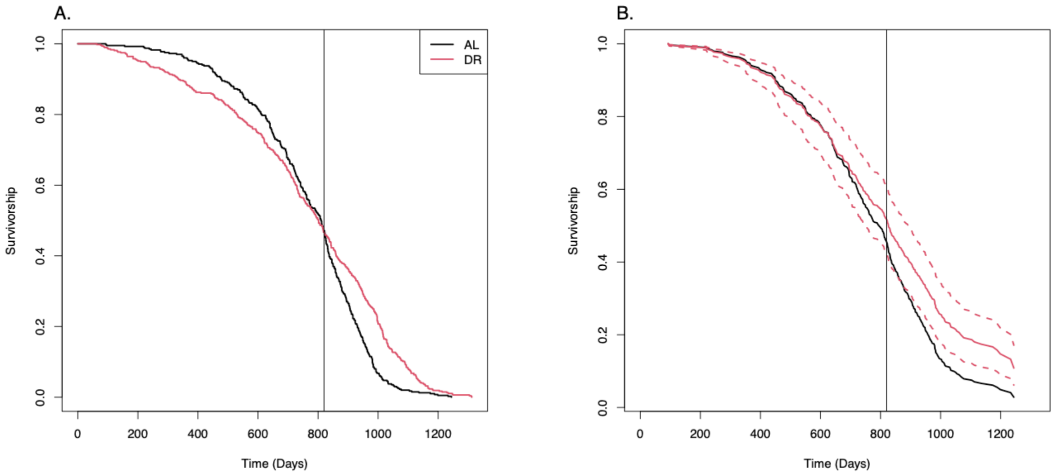

(A) Survival curves for all animals pooled together based on raw data. (B) Predicted survival curve for collected animals based on strain-specific estimates of deviation from baseline hazard and the lnHR from the CMM, and taking the collected AL data as the baseline hazard in a typical strain. Dashed lines are predictions using the upper and lower 95% confidence limits for the lnHR. Vertical line at 820 days for reference.

eFigure 3

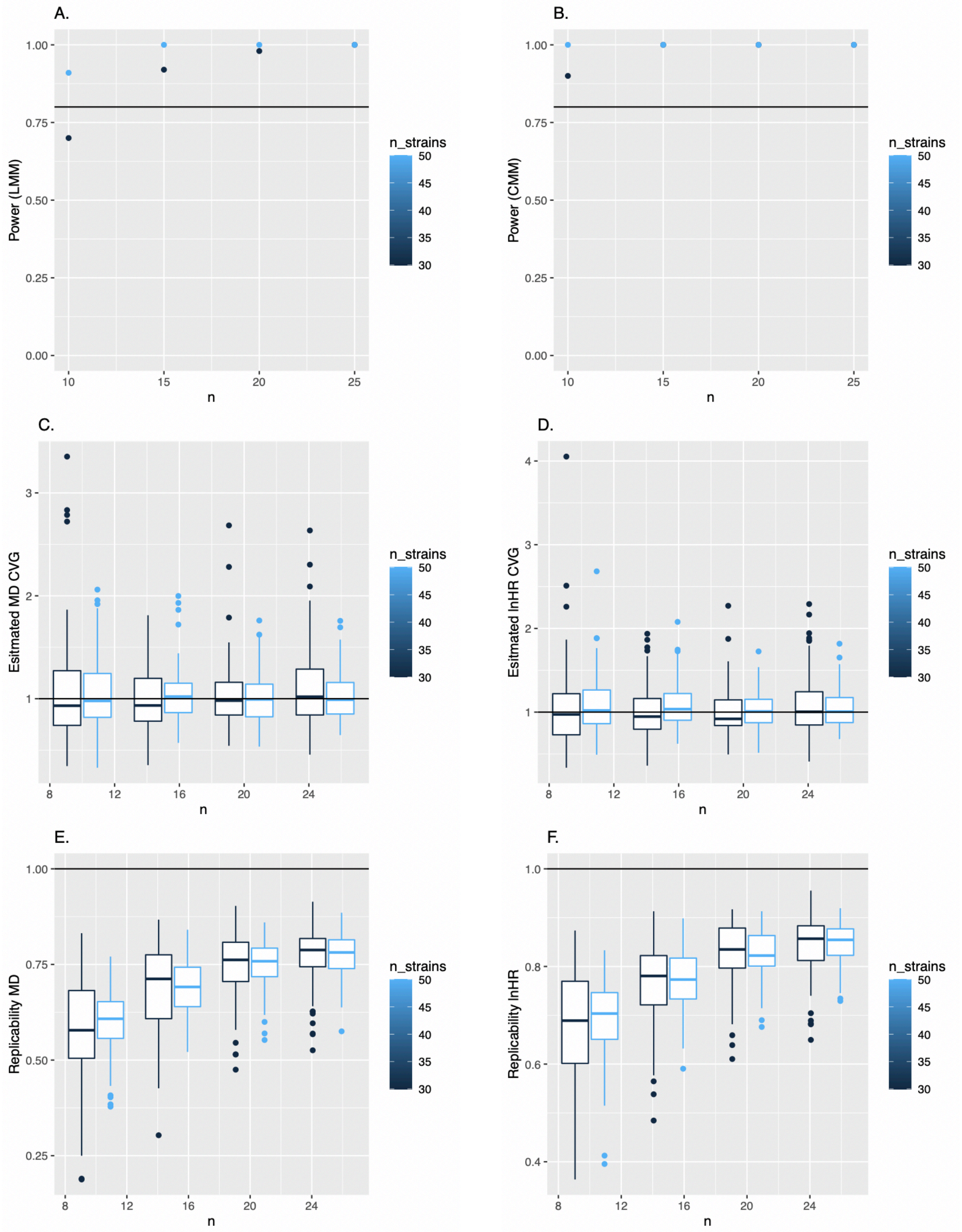

(A and B) Power to detect statistically significant variation in response to DR, in terms of mean difference in lifespan (MD) and mortality (lnHR) as a function of number of animals per group and number of strain (colour). Test is a LMM (MD) and CMM (lnHR) improvement of fit (Chi-square) with alpha = 0.05. (C and D) Estimated CV_G in response to DR, in terms of mean difference in lifespan (MD) and mortality (lnHR) as a function of number of animals per group and number of strain (colour). (E and F) Estimated replicability across two experiments of strain specific response to DR, in terms of mean difference in lifespan (MD) and mortality (lnHR) as a function of number of animals per group and number of strain (colour). Results are based on 100 replicate simulations per parameter set. Confirmation of favoured parameters with 10,000 replicates is recommended.
